# Unveiling diagnostic and therapeutic strategies for cervical cancer: biomarker discovery through proteomics approaches and exploring the role of cervical cancer stem cells

**DOI:** 10.3389/fonc.2023.1277772

**Published:** 2024-01-24

**Authors:** Ameneh Jafari, Masoumeh Farahani, Meghdad Abdollahpour-Alitappeh, Asma Manzari-Tavakoli, Mohsen Yazdani, Mostafa Rezaei-Tavirani

**Affiliations:** ^1^ Student Research Committee, Shahid Beheshti University of Medical Sciences, Tehran, Iran; ^2^ Skin Research Center, Shahid Beheshti University of Medical Sciences, Tehran, Iran; ^3^ Cellular and Molecular Biology Research Center, Larestan University of Medical Sciences, Larestan, Iran; ^4^ Department of Biology, Faculty of Science, Rayan Center for Neuroscience and Behavior, Ferdowsi University of Mashhad, Mashhad, Iran; ^5^ Laboratory of Bioinformatics and Drug Design, Institute of Biochemistry and Biophysics, University of Tehran, Tehran, Iran; ^6^ Proteomics Research Center, Shahid Beheshti University of Medical Sciences, Tehran, Iran

**Keywords:** proteomics, cervical cancer, biomarker, cancer stem cell, diagnosis, bioinformatics

## Abstract

Cervical cancer (CC) is a major global health problem and leading cause of cancer deaths among women worldwide. Early detection through screening programs has reduced mortality; however, screening compliance remains low. Identifying non-invasive biomarkers through proteomics for diagnosis and monitoring response to treatment could improve patient outcomes. Here we review recent proteomics studies which have uncovered biomarkers and potential drug targets for CC. Additionally, we explore into the role of cervical cancer stem cells and their potential implications in driving CC progression and therapy resistance. Although challenges remain, proteomics has the potential to revolutionize the field of cervical cancer research and improve patient outcomes.

## 1 Introduction

Cervical cancer (CC) is a major public health concern worldwide, ranking 4th in terms of incidence and mortality among cancers affecting women, accounting for approximately 570,000 new cases and 311,000 deaths annually (1). Human papillomavirus (HPV) infection is the primary cause of CC; however, smoking, age, and low socioeconomic status have been linked to the disease development (2, 3). Diagnostic tests such as Pap smears and viral DNA analysis, as well as the development of vaccines against different HPV genotypes, have all contributed significantly to reducing CC incidence (4). Despite advancements in screening and treatment, this cancer remains a major public health issue, particularly in low- and middle-income countries where access to cervical cancer screening is limited (5, 6). Generally, the process of CC encompasses multiple stages and involves the unregulated growth of cells. It begins with hyperplasia, advances to dysplasia, then develops into carcinoma *in situ*, and ultimately culminates in metastasis. According to studies, various forms of cancer exhibit similar genetic alterations, particularly in the signaling pathways responsible for regulating cell growth, proliferation, and apoptosis. The most significant changes are: (i) mutations in the mechanisms responsible for DNA maintenance and repair, which can occur in both somatic and germ cells, either through inheritance or sporadically; (ii) the conversion of proto-oncogenes into oncogenes as a result of mutations that modify the position, structure, expression, or function of genes; and (iii) mutations that inhibit tumor suppressor gene activity (7).

High-throughput techniques make it possible to assess cell physiology and the microenvironment in both normal and pathological circumstances (7). These methods also facilitate distinguishing between significant and insignificant cellular changes throughout disease progression and identifying the molecular characteristics of the disease at the genomic, transcriptomic, and proteomic levels (7). Proteomics, the large-scale study of proteins, has emerged as a powerful tool in cancer research for understanding the molecular mechanisms underlying cancer formation and progression. Proteomic analysis identifies and quantifies proteins in biological samples, providing insight into protein expression, post-translational modifications, protein-protein interactions, and signaling pathways (8, 9). Recently, there has been growing interest in applying proteomic techniques to cervical cancer research. Proteomics offers valuable insight into the molecular mechanisms underlying cervical cancer development and progression and has the potential to identify novel biomarkers for early detection and personalized treatment (10). Recently, proteomic approaches have been used for investigating molecular changes associated with CC. These studies have identified several proteins that are differentially expressed in CC compared to normal cervical tissue, including proteins involved in cell cycle regulation, DNA repair, apoptosis, and immune responses (11, 12). For instance, proteomic analysis of cervical cancer tissues recognized overexpression of the oncogene c-Myc (13), ZNF217 (11), and reticulocalbin 3 (RCN3) (14). These results suggest that dysregulation of these proteins may contribute to the development and progression of CC. Combination of proteomics data with other ‘OMICS’ datasets like genomics and transcriptomics has verified the role of systems biology in discovering potential biomarkers for personalized cervical cancer medicine.

This review aims to explore the current state of knowledge on cervical cancer through proteomics. It will discuss the latest findings on proteomic biomarkers for CC diagnosis and identifying new therapeutic targets. It will also summarize the challenges and limitations of proteomics in CC research, as well as potential future directions for this field.

## 2 Ideal biomarkers for cervical cancer

**Figure 1:**
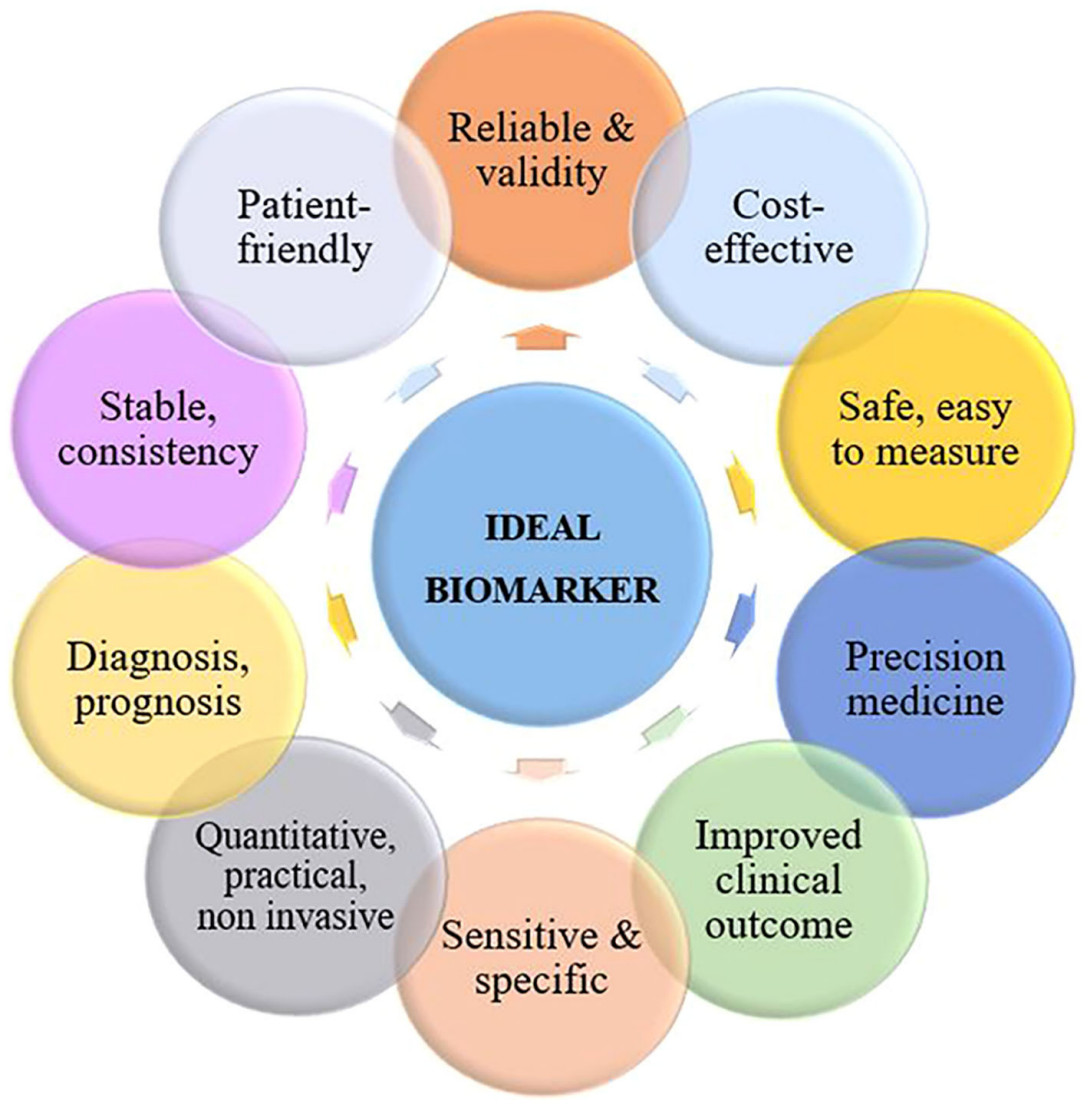
Characteristics of an ideal biomarker for cervical cancer.

Conventionally, CC is detected by Pap smear, colposcopy, and histopathological analysis. However, existing techniques have limitations, and researchers are actively characterizing new molecular biomarkers that hold potential for aiding in disease detection, risk assessment, treatment monitoring, and survival prognosis. Biomarkers are substances that discriminate between normal and pathological biological processes, such as diseases or tumors. These substances can encompass chemicals, proteins, or even segments of DNA and RNA. To be classified as a biomarker, a substance must be associated with a specific event, such as the diagnosis of a particular disease, disease progression, or the survival of a specific patient. Not all biomarkers exhibit equal effectiveness, but most do offer additional insights beyond what can be gleaned from clinical and pathological analyses alone. Ideally, biomarkers possess several common attributes as illustrated in **Figure 1**.

An ideal biomarker for CC should be specific to the disease and not present in healthy individuals or those with other diseases, as well as being sensitive enough for detection cancer at an early stage when treatment is most effective. It should be easily detectable from non-invasive samples such as blood or cervical swabs and provide accurate and reliable results consistently. Additionally, it should be cost-effective and readily available for usage in clinical settings, while also providing prognostic value for predicting the possibility of cancer recurrence or progression and informing treatment decisions.

A biomarker should also have a wide dynamic range for detection in order to accurately measure both high and low levels of the biomarker in various stages of the disease. Finally, an ideal biomarker should have good reproducibility and be able to improve patient outcomes by enabling earlier diagnosis and more effective treatment. It should also be easily understandable and patient-friendly to facilitate communication and decision-making between patients and healthcare providers.

## 3 Bioinformatics

Due to the development of omics technologies, a large amount of cancer-related data has been produced. The existence of this data and the multifactorial nature of cancer have raised the need for using bioinformatics to identify novel biomarkers. Numerous studies have been reported methods based on computational tools and integrated bioinformatics analysis to discover diagnostic and prognostic biomarkers in various types of cancer. One of the most potent computational techniques is meta-analysis, a statistical combination of results from multiple studies with a common hypothesis (15, 16). Many studies have used meta-analysis of cancer microarray data to identify differentially expressed genes and screen gene expression signatures in a single human cancer type or search for common transcriptional profiles among different types of cancer. RNA sequencing has also provided a powerful approach in many aspects of cancer study and enabled the characterization and classification of cancer and the detection of drug reactions. Some methods have also been developed to uncover functional roles of gene expression signatures, including Gene Ontology, biological pathway, and molecular networks analysis. The integration of gene expression signatures and protein-protein interaction network data is an effective approach to identify critical subnetworks and prioritize candidate disease genes. In recent years, cancer researchers have focused on data science and its application to cancer investigation. Machine learning (ML), a subset of artificial intelligence (AI), can be used to predict useful diagnostic and prognostic biomarkers. ML or AI methods can help in combining and integrating a large amount of cancer proteome data as well as overcoming challenges in data collection and analysis (17). Recently, AI approaches have been included in various researches to detect cancer stages and discover biomarkers. ML methods have received considerable attention due to their ability to predict diagnostic and prognostic biomarkers. For instance, Rader et al. provided a prognostic proteomic signature for cervical cancer and performed an ML technique for further exploration and validation (18). Due to the complexity of the causative factors, an accurate prognosis of cervical cancer is challenging. Hence, studies based on machine learning approaches have been developed to provide better models for predicting prognosis. However, reliance on public databases has limited the use of these efforts. Therefore, there is a need to investigate the value of ML in enhancing performance in predicting the prognosis of cervical cancer (19). Depending on the learning method, there are three broad categories of ML: supervised, unsupervised, and reinforcement learning. Supervised learning can be implemented to solve classification and regression problems, and as the most common category of ML in the medical field, it is mostly used for diagnoses and prognoses (20, 21). Unsupervised models are implemented for phenotyping of heterogeneous diseases (20, 22) and reinforcement models are used to optimize and maximize the desired results (20). Supervised algorithms of ML can improve the prediction of cervical cancer (23). Recently, deep learning (DL) models, as sub-discipline of the machine learning, have widely used to proteomics (24). DL models can be implemented in supervised and unsupervised settings (25). DL is far superior to traditional machine learning methods due to the use of raw data (26). For example, Dong et al. proposed a DL-based tumor classifier, which directly uses mass spectrometry raw data (24). DL methods have been used to analyze and integrate multi-omics data in precision medicine (25, 27). Studies indicate that research in the field of cancer diagnosis and treatment requires the integration of complex data (medical imaging, electronic health records, clinical, and omics data), which are suitable for DL. Deep learning methods provide promising approaches in effective modeling and integration of any type of omics and other data (25). Several studies have combined omics-based data and DL to better understand CC pathogenesis and improve cervical cancer diagnosis and characterization (28, 29). In their study, Long et al. analyzed eight distinct datasets containing genetic information from cancer, cervical intraepithelial neoplasia (CIN), and normal tissue samples using a systems biology approach to understand the multi-stage development of CC (28). Deep learning-based diagnostic models were created using specific genetic markers associated with the development of CC, and unbiased variable selection methods were employed. The authors also used survival analysis to identify potential biomarkers for predicting patient outcomes. The results indicated that the regulation of cell cycle, RNA transport, mRNA surveillance, and one-carbon metabolism through folate were crucial mechanisms involved in the initiation, progression, and spread of CC. Various combinations of genetic markers and ML techniques were effective in distinguishing CC from CIN and normal tissue across different datasets. Notably, a DL model utilizing 168 genes achieved an externally validated accuracy of 97.96% in differentiating cancer from normal tissue. Furthermore, the analysis revealed ZNF281 and EPHB6 as potential genetic markers for predicting the prognosis of cervical cancer. These findings provide new insights into the characteristics of CC and suggest that combining omics-based signatures with deep learning models could improve the diagnosis and management of cervical cancer in clinical settings (28).

## 4 Proteomics

Proteomics refers to the systematic and large-scale analysis of the proteome (proteins produced within an organism, system, or biological context). It involves the quantitative identification and analysis of proteins, taking into account post-translational modifications and alternative splicing that occur after protein production. So, proteomics studies not only scrutinize alterations in protein expression levels but also examine the post-translational modifications required to regulate protein function (9, 30). The primary goal of proteomics is to gain a comprehensive understanding of biological systems by examining all the proteins that comprise a cell. Comparative proteomics, an outlet of quantitative proteomics, focuses on analyzing changes in the proteome in response to environmental or disease stimuli. It is employed in the exploration and identification of biomarkers for various diseases (31).

During the last few years, proteomic approaches have been widely considered in CC research to identify potential biomarkers for early detection, diagnosis and prognosis of the disease, as well as to elucidate its underlying molecular mechanisms. Conventional protein analysis techniques such as one-dimensional sodium dodecyl sulfate-polyacrylamide gel electrophoresis (1D SDS-PAGE), Western blotting (WB), and ELISA, have limitations when it comes to comprehensive protein analysis and accurate quantification of protein expression levels. To address these limitations, two primary techniques are employed for large-scale comparative analysis: two-dimensional gel electrophoresis (2-DE) and Mass Spectrometry (MS). These techniques provide a more extensive and precise means of analyzing proteins and their expression levels. 2DE is a classic proteomic method that separates proteins based on their isoelectric point and molecular weight and MS is used to identify and quantify the separated proteins. MS acquires peptide spectral data and bioinformatics tools, such as MASCOT and DAVID Bioinformatics Resources, and network analysis, are then required for recognition the peptides and corresponding consensus proteins. They are then classified based on their function and subcellular location using human protein databases such as UniProt or Swiss-Prot (32).

**Figure 2:**
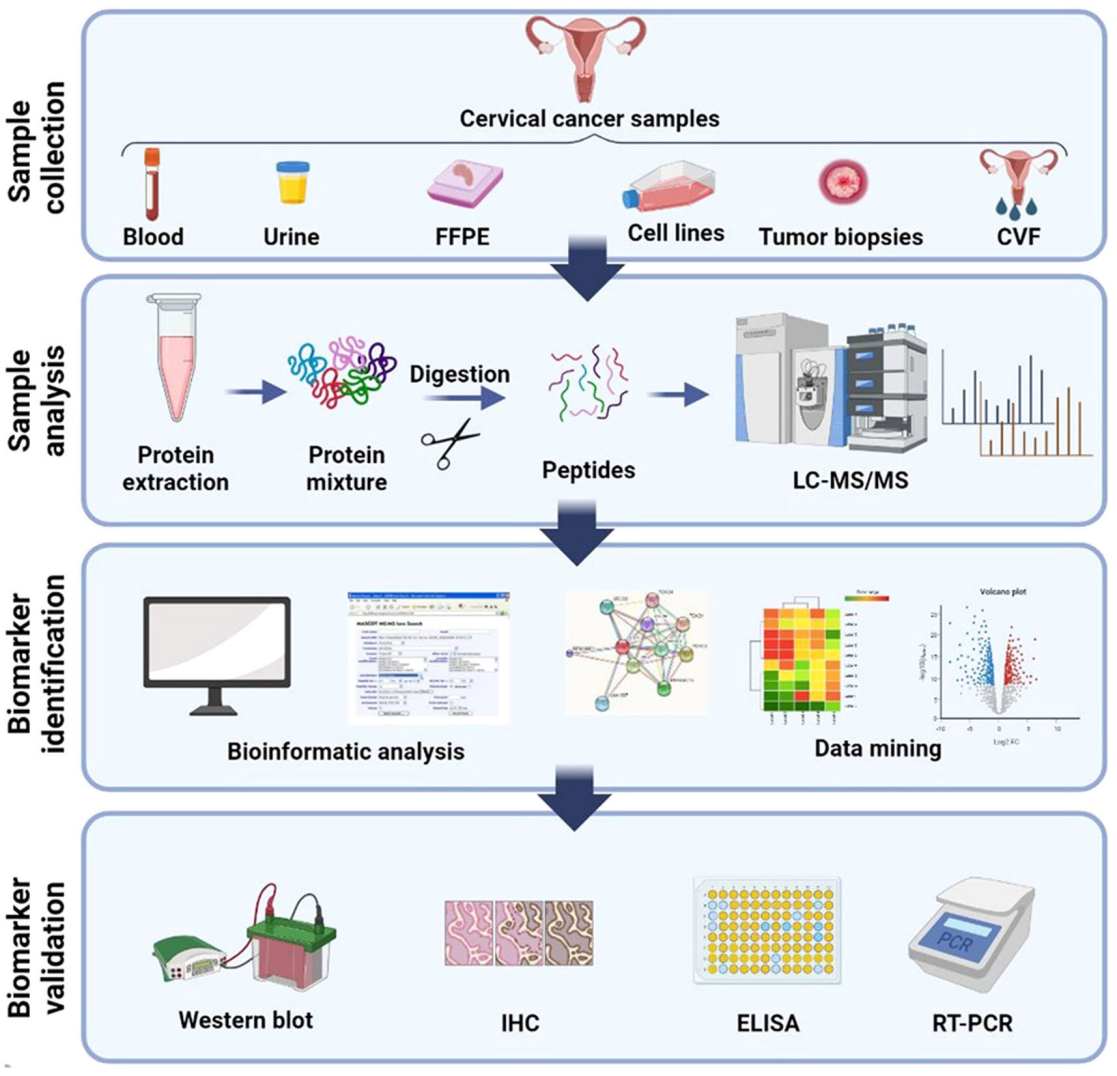
Schematic illustration of general method for proteomic analysis of CC derived from several samples. Blood (plasma/serum), urine, CVF (cervico-vaginal fluid), tissue, FFPE (formalin-fixed, paraffin-embedded), and cervical cancer cell lines are the several samples which proteomics studies have been assumed until now. Shotgun proteomics includes protein extraction from these samples followed by digestion, LC separation, and MS analysis. Bioinformatics and network analysis followed by target validation and biomarker discovery. Created with BioRender.com.

Generally, the process of discovering clinical biomarkers through proteomics involves two main phases. The first phase, known as the discovery phase, utilizes a shotgun approach to test and analyze a large group of protein biomarkers. The purpose of this phase is to identify potential candidates for further investigation. In the second phase, called the validation phase, a smaller group of selected candidates is validated using targeted proteomics methods in a blinded manner to confirm their efficacy as biomarkers (**Figure 2**).

To biomarker discovery for cervical cancer through proteomics, the exploration of various biological samples such as blood, cervicovaginal fluid (CVF), urine, and tissue becomes crucial. These samples play a pivotal role in uncovering potential biomarkers that can aid in the detection, diagnosis, and monitoring of cervical cancer. The use of proteomics, which involves the comprehensive analysis of proteins, enables researchers to identify and analyze protein profiles and alterations associated with cervical cancer. Unlike tissue or cytological samples, biological fluids such as urine, CVF, and blood fractions (serum or plasma) are more readily accessible (33). The accessibility and ease of collection of these fluid samples make them attractive options for biomarker research. By analyzing the protein composition and modifications within these samples, researchers can gain valuable insights into the molecular signatures and bimolecular changes associated with cervical cancer. The utilization of CVF as a body fluid sample in proteomic investigations offers distinct advantages. CVF is routinely used in screening programs, including high risk HPV (hrHPV) detection, making it a well-established and extensively studied biological specimen. Its significance extends beyond routine screening, as CVF can be utilized in research studies aimed at identifying novel biomarkers through proteomic approaches. The comprehensive analysis of proteins within CVF can unveil potential biomarkers specific to cervical cancer, providing valuable information for early detection, risk assessment, and personalized treatment strategies (34).

Plasma and serum from blood are commonly used for biomarker discovery in cervical cancer (35). These easily accessible and minimally invasive biological fluids contain important proteins. Previous studies have emphasized the role of plasma/serum-based proteomics and biomarker measurements in different cancer types (36, 37). However, challenges persist in implementing serum/plasma-based proteomics, such as study design, sensitive and reproducible sample preparation, and data analysis (37). The dynamic range of plasma/serum proteins poses an analytical challenge in proteomics, as low-abundance biomarkers can be masked by high-abundance common proteins (37, 38). Preanalytical variables, like freeze-thaw cycles and enzymatic degradation, can affect the stability and integrity of serum samples, potentially leading to protein degradation and loss of biomarkers. However, the addition of protease inhibitors during sample preparation can mitigate degradation. Despite these obstacles, recent advances in mass spectrometry-based proteomics have shown promise in cancer biomarker discovery from serum/plasma samples (37). These technologies enable quantitative analysis of proteins, providing a platform to study tumor-host interactions and monitor changes over time.

### 4.1 Proteomics-based biomarker discovery for cervical cancer

#### 4.1.1 2DE, 2D-DIGE

Two dimensional gel electrophoresis (2-DE) separates proteins according to their isoelectric point following separate these proteins based on their molecular mass on an SDS-PAGE gel, allowing for the high-resolution separation of complex thousands proteins in a single experiment.

Many studies used 2DE-MS to identify differentially expressed proteins (DEPs) in CC tissues compared to adjacent normal tissues. Proteomic biomarkers have the potential to transform CC diagnosis by providing a more reliable and accurate approach than traditional methods such as Pap smear and HPV testing. Numerous studies have found DEPs in CC tissues and body fluids that could be used as biomarkers for detection and diagnosis of this cancer.

Two-dimensional difference gel electrophoresis (2D-DIGE), a modified version of 2D-PAGE, uses three fluorescent tags of equal mass and charge to label different proteins, allowing the separation of three different protein samples in a single gel. Thus, gel-to-gel variability is avoided, offering greater sensitivity and reproducibility than conventional 2DE, facilitating comparison of sample proteomes and quantitative proteomics analysis (39).

In a study, combined laser capture microdissection and 2D-DIGE were performed to compare tissue from HSIL and cervical carcinoma with tissue normal cervical epithelium. Twenty-three statistically significant DEPs were identified, among them, cornulin, HSPB1, MnSOD, and PA28β were extra analyzed by IHC. Cornulin was distinguished as the most prominent putative biomarker (40). Lokamani et al. (41) analyzed serum samples of controls, and patients with CINIII and both early and late stages squamous cell carcinoma by 2D-DIGE/LC MS. Twenty DEPs were identified, of which four proteins namely complement factor H, ceruloplasmin, CD5-like antigen, and gelsolin further validated by ELISA. Biological network analysis revealed that ceruloplasmin and gelsolin are closely interacted with the oncogene NF-κb, they may serve as prognostic indicators for the development of high grade lesions to CC (41).

#### 4.1.2 MALDI

Lin et al. used two-dimensional difference gel electrophoresis (2D-DIGE) coupled to MALDI-TOF MS to compare proteome between the neuroendocrine and the nonneuroendocrine cervical cancer. They compared cervical cell line HM-1 of neuroendocrine origin with cell lines HeLa, ME-180, and CaSki of non-neuroendocrine origin and identified 82 DEPs. Results shown Transgelin and galectin-1 were overexpressed, whereas, stathmin and PGK-1 were underexpressed in the HM-1 cells in comparison to the cells of nonneuroendocrine origin. Further validation of finding proteins by WB was done in all cell lines, and finally, these proteins were implied as potential neuroendocrine biomarkers. In an *in vivo* study, 2D-DIGE approach was used to analyze tissue samples from CIN and cervical squamous cell carcinoma (CSCC) against normal cervical tissue specimens. Among 26 statistically significant DEPs, S100A9, PKM2, and eEF1A1 were proposed as putative diagnostic biomarkers after being validated by WB and immunohistochemistry (IHC) (42).

Using 2D/MALDI TOF MS, 30 DEPs were identified between cervical cancer tissues and normal control tissues, of which HSP60 was upregulated in cancerous samples (43).

In a similar study, seven proteins (AIF-1, ALP2, B-FABP, CDK4, ICA69, NCK- 1, and PRSS1) were upregulated in cancer samples, of which differential expression of B-FABP, CDK4, and NCK-1 was also validated by WB and IHC, and these could be used as potential cervical cancer markers (44). In the same context, the protein expression patterns of squamous cell cervical cancer (SCC) tissues were compared to normal tissues using 2D/MALDI TOF MS. A total of 35 DEPs were identified,17 proteins were overexpressed and 18 proteins were under expressed. 14-3-3ϵ, annexin A1, α-enolase, keratin, tropomyosin, and SCCA-2 were the most prominent findings (45).

Wang et al. analyzed tissue samples with and without pelvic lymph node metastasis using 2D-DIGE and MALDI-TOF/TOF MS techniques and MASCOT and the BioTools software. They detected three proteins, including FABP5, HspB1, and MnSOD related to metastatic processes in CC (46).

One study identified a panel of biomarkers consisting of three proteins (Mimecan, Actin from aortic smooth muscle and Lumican) that showed an increased expression and four proteins (Keratin, type II cytoskeletal 5, Peroxiredoxin-1 and 14-3-3 protein sigma) that showed a decrease in their protein expression level in cervical cancer in comparison with normal cervix cells (47). In another study, Guo et al. (48) used 2DE and MALDI-TOF-MS for screening and detecting cervical squamous cell carcinoma (CSCC). They screened 10 plasma proteins as candidate biomarker, mainly including lipid metabolism-related proteins (APOA1, APOA4, APOE), metabolic enzymes (MASP2, CP, F2), complement (CFHR1, EPPK1), immune function-related proteins (IGK@), and glycoprotein (CLU) (48). Network analysis indicated dysfunction of molecular transport, lipid metabolism, and small molecule biochemistry pathways in CSCC. Acute phase response signaling, IL-4 signaling and JAK/Stat signaling were known as the canonical pathways that are overrepresented in CSCC (48). Han et al. (49) using gene ontology (GO) and KEGG analysis revealed that 243 DEPs were mostly enriched in the cholesterol metabolism pathway, complement and coagulation pathway, the IL-17 signaling pathway, and the viral protein interaction with cytokine and cytokine receptor pathway. By validating the hub proteins using the ELISA, the authors introduced ORM1 (Orosomucoid 1) and APOF (Apolipoprotein F) as novel potential plasma markers in high grade squamous intraepithelial lesion (HSIL) and cervical cancer (49). Proteins such as Vimentin (50), COPA (51), eEF1A1, and PKM2 (42) were also considered as candidate biomarkers for early diagnosis of CC and new targets for therapy.

#### 4.1.3 LC-MS

Liquid chromatography, in combination with mass spectrometry (LC-MS), is the prevailing method employed to determine and measure the proteins present in a proteome. This highly sensitive technique is capable of identifying even the scarcest proteins, thereby ensuring an extensive and thorough analysis. Gu et al. (52) using liquid chromatography mass spectrometry analysis (LC-MS) found more than 200 proteins in high-grade dysplastic cells versus morphologically normal cervical cells with 3-fold difference in protein level. Interestingly, they observed significant overexpression of nuclear and mitochondrial proteins in HSIL specimens. Also, liquid chromatography coupled with tandem mass spectrometry (LC-MS/MS) is a broadly used proteomic method that separates proteins based on their physicochemical properties and identifies them using MS. Pappa et al. (53) in their paper describing the separation and enrichment of membrane proteins from three different CC cell lines, HeLa, C33A, and SiHa. They used LC-MS/MS to perform proteomic analysis on these cells, as well as bioinformatics analysis with Proteome Discoverer 1.4, SEQUEST, and UniProt. Using the Mann-Whitney statistical analysis, they were able to identify 263, 262, and 152 unique transmembrane proteins in C33, HeLa, and SiHa cell, respectively. CKAP5, CLPTM1, FAM120A, TMX2, and NCSTN were the greatest prominent DEPs in CC cell lines among the identified transmembrane proteins (53).

Researchers in one study combined laser capture microdissection and Nano LC- MS/MS to analyze protein levels in tissue sections from SCC patients compared to healthy tissue. Parallel Reaction Monitoring (PRM) was used to validate the differential proteins identified through the shotgun proteomics approach. They found increased levels of proteins in tumor tissue, including a significant increase in the MCM family of proteins and associated proteins. These MCM proteins play a critical role in DNA replication and their up-regulation is associated with tumor progression and early malignant transformation in various cancers. The article suggests that targeting MCM proteins could be a potential therapeutic strategy and that they may serve as molecular markers for cancer diagnosis. The authors used restrictive analysis techniques and identified a network of proteins related to “DNA Replication, Recombination, and Repair” that correlated with cervical cancer. Moreover, the study found specific proteins up-regulated in cervical cancer tissue and cell lines, such as CEACAM5 and S100P. The article also discusses the relevance of these findings to the development of diagnostic markers for cervical cancer and the potential use of these proteins in risk stratification and triage testing for HPV-positive women (10).

In another study, plasma samples from healthy individuals, low grade squamous intraepithelial lesion (LSIL), HSIL, CC, and post-treatment CC patients were analyzed using LC-MS/MS. The study identified five new protein biomarkers (AFM, CFI, F9, HPR, and ORM2) for cervical precancerous lesions and CC prognosis. LSIL and HSIL groups had nine differential proteins compared to controls, while CC group had five. ORM2 and HPR showed significant differential expression in LSIL and HSIL, indicating potential as cervical carcinoma biomarkers. F9 expression increased with lesion progression, suggesting a potential biomarker for CC. AFM and CFI protein levels decreased post-treatment, indicating predictive value for therapeutic efficacy. Enrichment analysis linked the DEPs to the complement system and coagulation cascades pathway (54). Recently, Aljawad et al. (55) analyzed the proteomes of cervical cancer tissue samples and normal cervical tissues using tandem mass tag (TMT) labeling and LC-MS/MS. They identified 336 differentially expressed proteins in cervical cancer tissues compared to normal tissues. Functional analysis of these proteins revealed their involvement in processes such as cell adhesion, cell cycle regulation, and DNA repair. The study also identified potential biomarkers for cervical cancer, including proteins such as KPNA2, DCN, MCM2, and COL1A1 (55).

#### 4.1.4 SELDI

Surface-enhanced laser desorption/ionization (SELDI) is another proteomic method that use for biomarker detection in cervical cancer. By SELDI-TOF-MS method in one study, 19 differentially expressed and statistically significant were detected between invasive cervical squamous cell carcinomas and normal cervix. A diagnostic model with two protein peaks at 3,977 m/z and 5,807 m/z was developed, with specificity 83.78% (31/37) and sensitivity 97.29% (36/37) (56).

#### 4.1.5 iTRAQ

Another quantitative proteomic approach is isobaric tags for relative and absolute quantitation (iTRAQ) that uses isobaric tags to label peptides, allowing for the relative and absolute quantification of proteins. A total of 3200 proteins were identified in 23 HPV +/HPV - clinical samples using iTRAQ peptide tag followed by LC-MS/MS. This is a proteome mining study that employs a new method to identify proteins, without the goal of comparing different groups and pinpointing the point of DEPs. Additionally, six HPV proteins were known and the presence of the corresponding viral DNA was proved by PCR method (57).

Recently, Xia et al. published a study that used iTRAQ-based quantitative proteomic analysis to examine the impact of metformin on migration and invasion of the CC cell lines. The mechanism by which metformin hinders CC cell propagation and invasion was investigated. The authors discovered 53 DEPs, 20 up-regulated proteins, and 33 down-regulated proteins after metformin treatment. Proteomic analysis, combined with tumor xenograft modelling, revealed that metformin reduced the expression of five proteins, namely TGFβ-1, TRIM26, CCPG1, MTR, LGMN, SLC38A2, ATP6AP1, CIRBP, and PTP4A1, while increasing the expression of IGFBP7 and CYR61. In this proteomic assay, the authors concluded that metformin could inhibit the proliferation and invasion of CC cells (58).

In a 2020 publication, Ma et al. conducted a proteomic study on cervical adenocarcinoma. They compared the proteome of normal cervical samples with individuals with endocervical adenocarcinoma using iTRAQ marking and LC-MS-TOF techniques (59). Cervical adenocarcinoma and endocervical adenocarcinoma are the two most common types of CC, with the latter being more common in young people. The study found a total of 711 proteins, with 237 proteins showing differential expression in endocervical adenocarcinoma and 256 of which showing differential expression between *in situ* adenocarcinoma and normal individuals. Additionally, 242 proteins exhibited distinct expression patterns between adenocarcinoma *in situ* and endocervical adenocarcinoma. According to a GO analysis of 1056 DEPs, the highest proportions were related to cellular processes, metabolic processes, response to stimuli, and biological regulation. The authors established that APOA1 could be a candidate marker for cervical adenocarcinoma and a research target for determining the disease’s functional mechanisms (59).

Han et al. used iTRAQ-MS on a panel of normal cervical tissues, HSIL, and CC tissues. They identified 72 DEPs both in CC vs normal and CC vs HSIL. The expression of HMGB2 was markedly higher in CC than that in HSIL and normal. The majority of proteins that identified in the study were those that bind to nucleic acids. Additionally, further studies revealed that HMGB2 was commonly up-regulated in cervical cancer samples and that this up-regulation was related to the size, depth, and FIGO stage of the initial tumor, resulting in tumor progression. These findings imply that HMGB2 may aid in the development of cervical cancer and that cervical cancer patients’ prognoses may be affected by the presence of HMGB2 in the tissues of the disease (60).

#### 4.1.6 Targeted proteomics

In recent years, technical advances in mass spectrometry-based proteomics has provided potential for use in profiling and deciphering the complexity of biological systems and understanding molecular mechanisms. Especially, clinical proteomics research has created the large-scale study of proteins and systems insights into disease understanding and biomarker discovery. Traditionally, a MS instrument uses a data-dependent acquisition (DDA) mode. In DDA-MS, a subset of the most abundant precursor ions is isolated based on the MS1 scan, and isolated ions are fragmented in sequential MS2 scans. When sampling breadth and discovery were the main goals, the DDA approach has been canonical for proteomics. However, the reproducibility of the DDA method is poor and there are challenges in detecting low-abundant ions (61–63). A high analysis throughput targeted proteomic approach with high sensitivity and absolute quantification efficiency that uses MS to quantify specific proteins of interest is SRM (selected reaction monitoring). In this sense, the SRM followed by LC-MS assay can be used for the routine screening of the HPV16 viral load in ThinPrep cervical smears for the early screening of cervical disease (64).

Multiple reaction monitoring mass spectrometry (MRM-MS) and parallel reaction monitoring mass spectrometry (PRM-MS) provides a highly sensitive method and reproducible quantitative detection of proteins, although these methods are not suitable for the discovery purposes and show limited throughput (61, 65). To tackle these challenges, data‐independent acquisition (DIA) methods have been developed that have the merits of both DDA and targeted methods, including high discovery power and high reproducibility, respectively. In contrast to DDA, where the mass spectrometer selects specific ions for fragmentation based on intensity, DIA fragments everything without selecting the intensity (61, 66).

**Table 1** shows the summary of studies on CC biomarkers discovered through proteomics.

**Table 1:** Putative cervical cancer biomarkers identified in different biological samples through proteomics analysis.

Candidate Biomarkers	Sample	Assay/technique	Conclusion	Reference
ATP6AP1, CIRBP, CYR61, CCPG1, IGFBP7, LGMN, MTR, PTP4A1, SLC38A2, TGF-1, TRIM26,	HeLa, SiHa	iTRAQ-MS, WB	Possible therapeutic target	(58)
CLPTM1, CKAP5, FAM120A, TMX2	HeLa, SiHa, C33A	LC-MS/MS	Possible therapeutic target	(53)
TIMP1, ADAM10, FUCA1, SOD2, NEU1	HeLa, SiHa, C33A	LC/MS-MS, WB, MRM	Possible therapeutic target	(67)
Cornulin	Tissue	2D, MALDI-TOF-MS, IHC		(40)
AIF-1, ALP-2, B-FABP, CDK4, ICA69, NCK-1, PRSS1,	Tissue	ESI-MALDI-TOF/MS, WB, RT-PCR IHC	Diagnostic marker	(44)
S100A9, eEF1A1, PKM2	Tissue	2D, MALDI-TOF-MS WB/IHC		(68)
FABP5, HspB1, MnSOD	Tissue	2D-DIGE MALDI-TOF/TOF MS	Diagnostic, prognostic marker	(46)
G6PD	Tissue	iTRAQ NanoLC-MS/MS qRT-PCR. WB Microrray	Possible therapeutic target,	(69)
VEGF, VEGF-C	Serum, tissue	IHC, ELISA	Prognostic marker	(70)
TKT, APOA, FGA	Serum	LC-ESI-MS/MS, ELISA	Prognostic marker	(71)
AACT, A1AT, TRFE, FETUA, KNG1, VTDB	Serum	iTRAQ- LC-MS/MS	Diagnostic marker	(72)
SCC-Ag, hs-CRP, CA-125	Serum	ELISA	Prognostic marker	(73)
APOA4, APOA1, APOE), EPPK1, CFHR1, CP, F2, MASP2, CLU	Plasma	2D-DIGE MALDI-TOF/TOF MS ELISA	Diagnostic, Possible therapeutic target	(48)
APOA1, mTOR	Plasma	2D HPLC, LTQ MS/MS	Potential biomarker	(74)
ASAH1, CYC, DDX5, ENO1, PCBP2, TYPH	CVF	iTRAQ-MS	Diagnostic, Prognostic marker	(75)
ACTN4, VTN, ANXA1, CAP1, ANXA2, MUC5B	CVF	LC-MS/MS	Potential biomarker	(76)
ACTN4	CVF	ELISA	Potential biomarker	(77)
CD44, LRG1, MMRN1, S100A8, SERPINB3,	Urine	LC-MS/MS WB	Diagnostic marker	(78)
APOA1, MPO	cervical mucus	LC-MS/MS	Diagnostic marker	(59)

### 4.2 Response to treatments

Another application of proteomics studies of CC is the study and analysis of proteins involved in chemotherapy or radiotherapy resistance. In this regard, a recently published paper on radioresistance predictive models based on a protein panel created from 181 samples of patients with advanced CC. The authors discovered that proteins BCL2, CD133, CAIX, ERCC1, and HER2 are predictors of survival in advanced CC patients after a reverse-phase protein assay in tumor samples and validation by Western blot (WB), which may be useful in finding responses to chemoradiation (79).

Proteomics approaches have also been used to evaluate the effects of drug and understanding their mechanisms in CC. To clarify the antiproliferative mechanisms of the widely used drug cisplatin in combination with radiotherapy, a proteomic research was done on treated and untreated HeLa cervical cancer cells. Based on the results, the expression of I-TRAF and p27Kip was increased in the cisplatin-treated group, while c-myc and PCNA were downregulated in the similar group in comparison to untreated cells. Considering that the involvement of I-TRAF and other known downstream targets in apoptosis was confirmed by WB, apoptotic pathways are probably the mechanism of action of cisplatin.

Li et al. (80) have recently demonstrated a study to identify glycopeptide markers in serum glycoproteins that can predict the efficacy of chemotherapy for locally advanced cervical cancer (LACC), thus enabling personalized treatment. They conducted a comprehensive screening of site-specific N-glycopeptides in serum samples from LACC patients before and after neoadjuvant chemotherapy (NACT). Liquid chromatography coupled with high-energy collisional dissociation tandem mass spectrometry (LC-HCD-MS/MS) was used for quantitative analysis and differential glycopeptides in chemo-sensitive and chemo-resistant patients were identified. The study identified 148 glycoproteins, 496 glycosylation sites, and 2279 complete glycopeptides in serum samples from LACC patients. Before and after chemotherapy, the NACT responsive group showed 13 differentially expressed glycoproteins, 654 differentially expressed glycopeptides, and 93 differentially expressed glycosites, while the NACT nonresponsive group showed 18 differentially expressed glycoproteins, 569 differentially expressed glycopeptides, and 99 differentially expressed glycosites. Six glycopeptides MASP1, ATRN, LUM, CO8A, CO8B, and CO6 were identified as biomarkers for predicting the sensitivity of neoadjuvant chemotherapy in LACC. High levels of these glycopeptides indicated chemotherapy effectiveness. The authors suggested that theses six N-glycopeptides may serve as potential biomarkers for predicting the efficacy of chemotherapy in cervical cancer (80).

Using a 2D/MALDI TOF MS proteomics technique, researchers examined the impact of neoadjuvant chemotherapy (NAC) involving paclitaxel and cisplatin (CP) on cancer tissues before and after treatment. They observed that a group of 13 proteins showed varying levels of expression between the two groups. Specifically, two proteins were found to be upregulated, while eleven proteins exhibited the opposite trend in the post-NAC group compared to the pre-NAC.

Among them, HSP27, HSP70, ALDA, and ENO1 were chosen for additional confirmation by immunoblotting. This research has potential to contribute to the identification of biomarkers that could be valuable in monitoring the response of patients to chemotherapy (81).

In a similar study by same researchers, the synergistic effect of 5-fluorouracil (5-FU) and CP was assessed by a 2D gel method on the HeLa cells. Four different sets, including untreated cells, treated with 5-FU, treated with CP, and treated with a combination of 5-FU and CP were used. As shown by WB analysis, the expression of downstream targets involved in apoptosis was observed to be at its highest level in treated cells with both drugs, as I-TRAF and CIDE-B were upregulated, whereas c-myc and SCCA-2 were downregulated in this group. This study shows the synergistic effect of the above drugs by activating intrinsic and extrinsic apoptosis pathways (mitochondria-mediated apoptosis pathway and membrane death receptor-mediated apoptosis pathway) (82).

In a proteomics study on cervicovaginal fluid (CVF) samples, 12 DEPs were detected in the CVF obtained from healthy and precancerous clinical samples using HPLC coupled with MALDI-TOF MS. Among these DEPs, Alpha-actinin-4 was selected for additional validation using ELISA, and it was suggested as a potential biomarker for distinguishing between healthy individuals and those in precancerous stages (77).

In another proteomic analysis, 2D/MALDI TOF MS was used to investigate the effects of suberonylanilide hydroxamic acid (SAHA) on cervical cancer cells. SAHA is a histone deacetylase inhibitor that has shown promise as a potential treatment for various types of cancer, including cervical cancer. The researchers treated CC cell lines with SAHA and analyzed the proteome of the treated cells using quantitative proteomic techniques. They identified nine DEPs in SAHA-treated cervical cancer cells compared to control cells. Functional analysis of these proteins revealed their involvement in processes such as protein metabolism, molecular chaperone, transcription, carbohydrate metabolism, apoptosis and/or anti-proliferation cellular processes. The results of this study support the hypothesis that SAHA is a potential inhibitor of cervical cancer. The study also identified potential biomarkers for SAHA treatment response, such as the PGAM1.

### 4.3 Challenges of translating proteomics data into clinic

Biomarker discovery for cervical cancer by proteomics has several limitations. One of the main limitations is the technical challenges in sample storage and preparation, which may affect the comparability of proteomics analyzes. In addition, standardized procedures for sample collection, processing, and analysis should be built up to guarantee reproducibility and comparability of results across studies. Furthermore, validation studies on larger and more diverse patient cohorts are needed to confirm the diagnostic and prognostic value of the identified biomarkers. Low disease prevalence and heterogeneity of diseased tissues are often not considered in biomarker studies, leading to potential issues in study design. In addition, concealing of low-abundance proteins by high-abundance proteins in blood and urine samples can hinder the distinguishing proof of biomarkers. Furthermore, the complexity of the proteome and the variety of molecular events underlying clinical observations make it challenging to completely understand the molecular mechanisms of CC.

One of the obstacles in making proteomics applicable in clinical settings is the cost and the advanced expertise it demands. Nevertheless, presently, state-of-the-art mass spectrometers are priced similarly to high-end imaging equipment that is commonly employed in oncology. It could be argued that with dependable instrumentation and consistent usage, the expenses might even be lower than those of other technologies. Finally, integrating proteomic biomarkers into current screening and treatment protocols requires a multidisciplinary approach involving clinicians, pathologists, and researchers. However, there is still a need for knowledgeable operators and analysts to handle the instruments and interpret the data. It is hoped that improved instrumentation, further technical development, extensive training, standardization and a problem-oriented approach will eventually reduce the problems for the discovery of biomarkers for cervical cancer.

## 5 Cervical cancer stem cells

### 5.1 Cancer stem cells

Cancer stem cells (CSCs) are a small specialized subpopulation of malignant cells that possess unique characteristics such as self-renewal, ability to differentiate into various cell types, anchorage independent growth, metastatic potential, and resistance to treatment (83). The concept of CSCs is supported by growing evidence, with these cells being identified in various types of cancer, including acute myeloid leukemia, hematologic malignancies, and solid tumors (84). However, the exact origin of CSCs is still not fully understood and two main hypotheses have been proposed. According to one hypothesis, CSCs arise from differentiated non-stem cells that acquire stem cell-like properties after undergoing malignant transformation (85). This process is influenced by epithelial-mesenchymal transition (EMT), which can trigger stem cell features like self-renewal and expression of stem cell-related markers (84). The second hypothesis suggests that non-malignant stem cells undergo transformation into CSCs due to oncogenic somatic mutations (84, 86). Some studies indicated that CCSCs exhibit features of both mentioned hypotheses (87).

It is believed that CSCs are crucial in the growth and expansion of malignant tumors, as they may be responsible for tumor initiation, growth, and metastasis. They make up only a small fraction of tumor cells, ranging from 0.1% to 10%, and express lower levels of tumor-associated antigens compared to other tumor cells (88). Following cytotoxic treatments, there is an enrichment of cancer stem cells in residual tumor cells, suggesting their role in chemoresistance and disease relapse. Cancer stem cells may evade chemotherapy and apoptosis while hijacking the host immune system, leading to aggressive tumors with poor prognosis (89). Although they represent a small proportion of the tumor, CSCs actively adapt to changes in their environment during cancer development and resistance to therapy. Therefore, investigating the molecular signature of CSCs in CC could provide valuable insights into developing effective therapeutic strategies for the disease (88, 90). A plethora of the studies have recognized a population of CSCs in CC that show characteristics of both cervical cancer cells and normal cervical stem cells.

### 5.2 Proteomics and CSCs

Clinical studies have consistently shown that the identification and validation of specific markers associated with cancer stem cells is crucial. The continuous advancements in genomic and proteomic studies have had a profound impact on biological research by facilitating the identification of distinct genes, proteins, and signaling pathways. These techniques play a significant role in differentiating the behavior of cancer stem cells from that of other cells, offering valuable insights into their unique properties. Identifying the novel candidate biomarkers related to cancer stem cell subpopulation and increasing knowledge about their biology and active pathways is of clinical relevance (91, 92). Genome data can be used to infer molecular perturbations in malignant cells, but do not reflect the protein function-related processes. Proteomics has greatly contributed to our understanding of CSCs. It has provided valuable insights into the molecular characteristics and functional properties of CSCs. By employing techniques such as mass spectrometry and protein profiling, researchers can identify and quantify proteins that are specifically expressed or modified in CSCs compared to non-CSC counterparts. This enables the discovery of CSC-specific protein markers and signaling pathways that govern their unique properties (92). The yielded knowledge has significant implications in clinical diagnosis, cancer classification, and prognosis prediction. Proteomic analysis also helps uncover CSC heterogeneity, plasticity, and drug resistance, providing insights into tumor progression and therapeutic strategies. Additionally, proteomics aids in identifying potential therapeutic targets for CSCs (93–95). For instance, Morisaki et al. performed proteomics analysis of gastric cancer stem cells to identify novel biomarkers for clinical diagnosis. They validated the identified candidate proteins by immunohistochemical analysis of 300 gastric cancers. The result of the study provided evidence that eight proteins (ALDOA, DCTPP1, GLG1, HSPA4, KRT18, VPS13A and RBBP6) may be potential cancer stem cell markers of gastric cancer. Among the eight candidate proteins, RBBP6 was suggested as a promising prognostic biomarker for gastric cancer (93). In another study, Bonardi et al. applied LC-MS/MS technology to analyze the plasma membrane proteome of leukemic stem cell-enriched fractions of two different acute myeloid leukemia samples. Combination of the proteomics with transcriptomics methods indicated eight subgroups of acute myeloid leukemia based on their specific plasma membrane expression profile (94).

### 5.3 Cervical cancer stem cells

**Figure 3:**
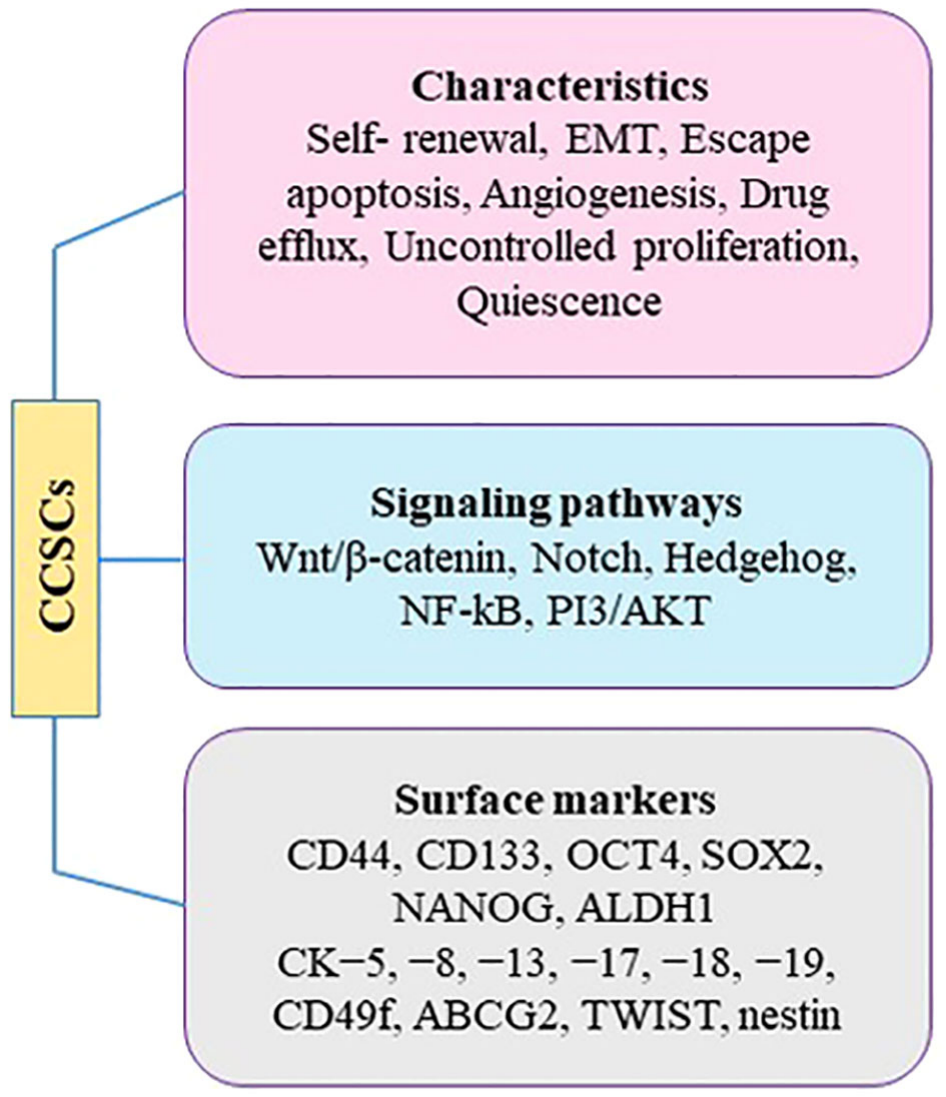
Characteristics of CCSCs.

Based on the theory of “clonal evolution” in carcinogenesis, CC arises from a breakdown of regulatory mechanisms that results in uncontrolled cellular growth in cells sharing similar molecular features (96). However, there is growing evidence of intratumoral heterogeneity in CC, which may be due to the existence of cervical cancer stem cells (CCSCs). These cells have several properties including, self-renewal, uncontrolled proliferation, efflux drug, and quiescence (**Figure 3**).

Studies suggest that CCSCs originate from non-malignant cervical stem cells present in the normal cervix. It proposed that these non-malignant stem cells undergo genomic instability and accumulate somatic mutations over time, particularly under the influence of hrHPV genes. Eventually, these non-malignant cervical stem cells transform into CSCs. During this transformation, functional changes occur in cervical stem cells, characterized by a decline in their original non-malignant stem cell properties, such as high proliferation and low differentiation, and a progressive increase in cancer stem cell properties like EMT, invasion, and metastasis. Recent studies support these hypotheses by observing significant enrichment of a hypoxia signature in CCSCs, as hypoxia promotes EMT and facilitates tumor cell invasion and metastasis (87). Indeed, slow-cycling CCSCs reside in niche zones of tumors and can initiate and sustain neoplastic growth, and even cause distant metastasis (97). Analysis of hallmark pathways revealed the upregulation of EMT-promoting pathways in CSCs. The study also observed changes in glucose metabolism, with increased glycolysis in CSCs associated with EMT and enhanced oxidative phosphorylation (OXPHOS) in highly proliferative non-malignant cervical stem cells (98). The transformation process was further characterized by the gradual enhancement of EMT properties in CSCs. Despite the role of HPV-induced oncogenesis in CC, there is still limited knowledge about the specific cell types involved in the development of the disease.

**Figure 4:**
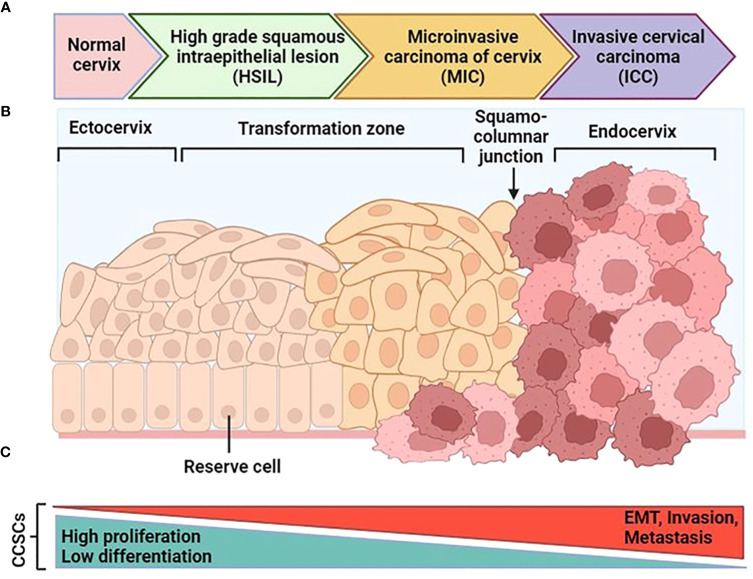
Schematic progression from normal cervical tissue to invasive cervical cancer. **(A)** After an hrHPV infection, tissue can progress to cervical intraepithelial neoplasia (CIN). After that it can progress to high-grade squamous intraepithelial lesions (HSIL) and early-stage cervical cancer as microinvasive carcinoma (MIC), and then invasive cervical carcinoma (ICC). **(B)** The normal cervical epithelium morphology is shown in the left followed by intraepithelial lesions and cervical cancer. **(C)** The functional changes in CCSCs during malignant transformation are characterized by two major features: the original non-malignant stem cell properties characterized by high proliferation and low differentiation gradually diminish, and the cancer stem cell properties characterized by EMT, invasion, and metastasis progressively increase. [Modified from Ref (87)]. Created with BioRender.com.

The cervix is a complex organ with different types of epithelium, including stratified epithelium in the ectocervix, single columnar epithelium in the endocervix, and transformation zone epithelium (99, 100) (**Figure 4**). The transformation zone is the primary site of cervical cancer origin. Traditionally, it has been proposed that cervical reserve cells, stem cell-like cells located near the squamous columnar junction (SCJ), are responsible for cervical cancer development (101). However, recent studies suggest that a discrete population of cuboidal cells in the SCJ may be the origin of cervical cancer.

While CSCs express certain cell surface markers, there is no universal collection of biomarkers that can be used to specifically identify and isolate CSCs (102). This principle also applies to identifying markers for CCSCs, as there are variations observed between tumors. Consequently, cervical cancer cells expressing a single stem cell marker may not always meet the criteria for being classified as CCSCs. Therefore, to isolate CSCs within a specific tumor location and across multiple tumor sites, it is necessary to employ a range of cell surface markers and functional indicators. Nevertheless, ongoing research is focused on discovering and exploring novel markers to enable diverse therapeutic options to cure CC. In this context, Zhang et al. (87) examined the expression of biomarkers in reserve cells and SCJ cells, both in the epithelial and tumor cell clusters. They found that CSCs exhibited high expression of reserve cell markers (KRT17, MKI67, and TP63) and low expression of SCJ cell biomarker (AGR2, CK7, GDA, MMP7, and CFTR) in CSCs, suggesting that the long-presumed reserve cells could indeed be the stem cells observed in the study. The authors suggested these specific markers for cervical CSCs, as potentially targets for future therapies (87).

### 5.4 CCSCs and therapeutic implication

Targeting CSCs is a promising therapeutic strategy that aims to eliminate cancer development and minimize recurrence. In this context, many pre-clinical and clinical studies have been demonstrated using different therapeutic agents against CSCs. Identification of molecular pathways, miRNAs and regulatory niches of CSC function can effectively suppress chemotherapy resistance of CSCs by using anti-CSC agents (103, 104). While there is limited data confirming the clinical diagnostic value of CCSC biomarkers, evidence suggests these biomarkers are not very sensitive and may be more indicative of lesions that have already progressed. However, several biomarkers, especially in combination, can be used for CC screening.

Chemotherapy and radiotherapy have shown effectiveness in enhancing the overall survival (OS) rates of CC patients. However, the presence of chemotherapy- and radiotherapy-resistant CCSCs contributes to disease recurrence and reduced OS. Because of their capacity to initiate tumor formation, CSCs have the potential to contribute to the process of cervical carcinogenesis, resulting in the occurrence of distant metastasis. Hence, specific targeting of CSCs can be a potential tool to prevent chemotherapy/radioresistance and reduce the risk of distant metastasis, secondary tumor generation, and tumor recurrence, thereby increasing the chance of CC patient survival (104, 105). To date, a series of specific chemicals have been shown to be effective in treating CCSCs. For instance, Yang et al. (106) observed suppression of proliferation, colony formation, migration, EMT, and differentiation and induction of apoptosis of HeLa cancer stem cells after doxycycline treatment. They also observed the decrease of stem cell markers such as SOX-2, NANOG, OCT-4, NOTCH and BMI-1 in these CCSCs. In addition, the authors showed through immunohistochemically assays that the stem cell marker SOX-2, the proliferation marker PCNA, and Ki67 were all significantly decreased in doxycycline-pretreated xenografts. In fact, doxycycline can reduce the tumor growth capacity of HeLa-CSCs *in vivo* (106).

Morusin, a prenylation flavonoid compound, demonstrated significant effectiveness in suppressing platelet aggregation, exhibiting antimicrobial properties, and reducing the production of superoxide anions (107, 108). Notably, it has cytotoxicity against certain types of human cancers, including breast cancer, colorectal cancer, and hepatocarcinoma (109, 110). Wang et al. assumed that morusin has a strong inhibitory effect on the growth and migration of human CCSCs, and its mechanism of action may involve the attenuation of NF-κB signaling, leading to the induction of apoptosis (111).

Phenethyl isothiocyanate (PEITC), a compound found in cruciferous vegetables, has been also investigated on cervical cancer stem cells for its potential anticancer properties (112–114). The researchers aimed to understand the molecular mechanisms underlying the anticancer effects of PEITC on HeLa cancer stem cells. They found that exposure to PEITC led to increased oxidative stress in CCSCs. Furthermore, the study revealed that PEITC treatment suppressed the activity of a transcription factor called Sp1 in cancer stem cells. Sp1 is known to regulate the expression of genes involved in cell growth, survival, and differentiation. The suppression of Sp1 by PEITC suggests that it may contribute to the inhibition of CCSC function (113).

Agarwal et al. in their study explored the mechanism of action of zoledronic acid in inhibiting the growth of cancer stem cells derived from cervical cancer (115). Zoledronic acid is a bisphosphonate drug commonly used for the treatment of bone diseases, including cancer-induced bone loss (116). The study found that zoledronic acid treatment effectively reduced the stemness phenotype of CCSCs (115). Zoledronic acid also induced apoptosis through the activation of two signaling pathways; the Erk1/2 pathway and the Akt pathway. The Erk1/2 pathway is involved in regulating cell proliferation and survival, while the Akt pathway plays a crucial role in cell survival and resistance to apoptosis. By inhibiting these signaling pathways, zoledronic acid effectively suppressed the growth and survival of CCSCs. This suggests that zoledronic acid may have potential therapeutic benefits in targeting cancer stem cells and inhibiting tumor progression (115).

Despite the therapeutic effects of those chemical agents, few drugs and molecular agents have been developed that specifically target CCSCs. By identifying CCSCs and gaining a better understanding of their surrounding microenvironment, it becomes possible to develop targeted pharmacological approaches tailored to these specific cells (104). Currently, several studies are exploring new target genes, signaling pathways, and proteins involved in the stemness of CC cells. CSC-specific markers, such as CD133 and CD49f, and signaling pathways, including PI3K/Akt/mTOR, Hedgehog, Notch or Wnt/β catenin, have been mostly used as therapeutic targets (117) (**Figure 3**). Another effective therapeutic approach is targeting of CSCs with nanoparticles (NPs). NP-based therapies have been used for targeting stem cell-specific signaling pathways and subsequently inhibition of stem cell-related functions. In this light, salinomycin NPs have been used for targeting CCSCs. Gelatinase-stimulating nanoparticles loaded with sal-doctaxel can be a promising strategy for increasing bioavailability, reducing side effects, and thus increasing antitumor effects by simultaneously suppressing CCSC and non-CCSC cells (118–120).

### 5.5 Biomarkers in CCSCs

Cancer stem cells have been characterized by certain specific markers, including CD44, CD90, CD133, CD271, aldehyde dehydrogenase 1 (ALDH1), and epithelial cell adhesion molecule (117, 121). However, there is no universal set of biomarkers that can be used to recognize and isolate CSCs CD44 and CD133 transmembrane glycoproteins are widely putative as general CSC markers in many types of tumors, and are involved in normal cellular processes and also in cancer development (122). They can be used as surface markers for isolation numerous types of CSCs, such as gastric colorectal, pancreas, breast, prostate, and cervical cancer (117). Characteristic expression profiles and cell surface markers of CCSCs make possible their isolation, evaluation, and directed targeting.

Cytokeratin (CK) and CDs are among the main proteins expressed in CCSCs. The proteins expressed in reserve cells and the immature squamous metaplastic cells of the cervix are CK -5, -8, -13, -17, -18, and -19. The expression of CK19, a biomarker associated with CCSCs, is meaningfully elevated in CC compared to individuals with benign lesions (123). CK8 and CK17 were detected in both CIN and CC tissues. CK17, in particular, has been linked to metastatic processes and the progress of extremely malignant diseases. Therefore, CK17 and CK19 can be regarded as biomarkers for CCSCs. Another protein, CD49f, is highly expressed in CCSCs and can be advantageous in their identification and isolation. Analysis of surface markers in sphere cells derived from different CC cell lines showed an increase in the population of CD133- and CD49f-positive cells compared to the monolayer cells (117).

Liu and Zheng (124) discovered that CCSCs exhibit elevated levels of ALDH1, which is associated with their differentiation potential, self-renewal, and tumorigenicity, similar to other CSCs. This led them to propose ALDH1 as a potential CSC marker. In an *in vitro/in vivo* study demonstrated by Ortiz-Sánchez et al. (125) putative CCSCs displayed distinct phenotypes, including CK17, CD49f+, AII+, p63+, and ALDH. Sphere culture presented a stemness characteristics considered by the presence of NANOG, β-catenin, and OCT4. The presence of CD49f and AII was related with the possibility of hrHPV infection in healthy cervical cells. The authors also demonstrated that ALDH ^bright^ cells had a higher tumorigenic capacity compared to ALDH ^low^ cells.

A study investigating the correlation between CCSC markers and the prognosis of CIN patients used immunohistochemistry (IHC) and RT-PCR techniques. The investigated markers were CD49f, SOX2, ALDH1, and musashi RNA binding protein 1 (MSI1). The results demonstrated that patients with high MSI1 expression and low CD49f expression had the poorest prognosis in CC. In contrast, tumors lacking MSI1 upregulation and CD49f expression had the most favorable prognosis. These results provide crucial clinical evidence linking CCSC markers to patient prognosis, highlighting the role of CCSCs in cancer progression and their significance as potential targets for therapy and prognostic indicators (126).

Other DEPs in CCSCs are Nucleostemin, TWIST, nestin, BMI1, piwi-like RNA-mediated gene silencing 2 (PIWIL2), TIMP metallopeptidase inhibitor 4 (TIMP4), LGR5, OCT, and SOX (117). Several studies investigated the role of different markers, including NANOG, Nestin, and MSI1, in the development and progression of CC and the regulation of CSCs. They found that these markers were highly expressed in CC and advanced stages of CIN, but had low expression in early stages and normal cervical tissue. However, there was no correlation between their expression levels and cervical cancer prognosis. The studies also revealed the involvement of these markers in other types of cancer (127, 128). The role of PIWIL2 in CC tumorigenesis was evaluated by Feng and colleagues (129). They observed expression of PIWIL2 in HPV+ CC cell lines but not in HPV- cancer cell lines. Knockdown of PIWIL2 decreased the proliferation, tumorigenic, and chemoresistant capacity of CC cells, while overexpression of PIWIL2 activated tumor-initiating capabilities and upregulated several cell reprogramming factors. The authors also confirmed that PIWIL2 is necessary in the transformation of cervical epithelial cells into CSCs, and it blocked the expression of P21 and P53 in CC cells, inducing cervical carcinogenesis.

In their study, Lizarraga et al. (130) established the role of TIMP4 in the stemness of CC cells. In animal models, overexpression of TIMP4 in CC cells led to faster tumor formation, activation of NF-κB signaling pathway, and an increase in CSC population with high expression of pluripotency markers as well as EMT markers and drug efflux transporters markers. Fahmi et al. (131) conducted a systematic review and meta-analysis of the CCSC markers to predict the OS and disease-free survival (DFS). They found that high expressions of SOX2, OCT4, ALDH1, CD44 and CD49f were associated with poor OS, and overexpression of the latter three was also associated with worse DFS. These findings propose that CCSC markers expression may assist the clinicians in the management or assessment of the CC status after surgery.

Recently, Cao et al. (132) performed a quantitative proteomic analysis to discover the alterations between parent cells and cancer stem-like spheroid cells in endometrial cancer (EC). They identified a 167 overlapped DEPs of two cell populations, 124 were down- and 43 proteins up-regulated in spheroid cells comparing with parent cells. The role of HIF-1 pathway was confirmed in spheroid cells by KEGG analysis. Consistent with proteomic results, elevated expressions of PFKFB3, GPRC5A, and HK2 of HIF-1 pathway was confirmed by qRT-PCR and WB in spheroid cells. HK2 promoted cancer stemness in EC. The principal role of LGR5 in CCSCs for the activation of Wnt/β-catenin signaling pathway is reported (133). The study showed that overexpression of LGR5 induces CSC features, including tumorsphere formation, chemoresistance, increased tumorigenic capacities, increased cell migration and invasion, and upregulation of stem cell-associated transcription factors. LGR5 overexpression in CC cells was also correlated with elevated expression levels of NANOG, OCT4, BMI1, and KLF4 (133).

**Figure 5:**
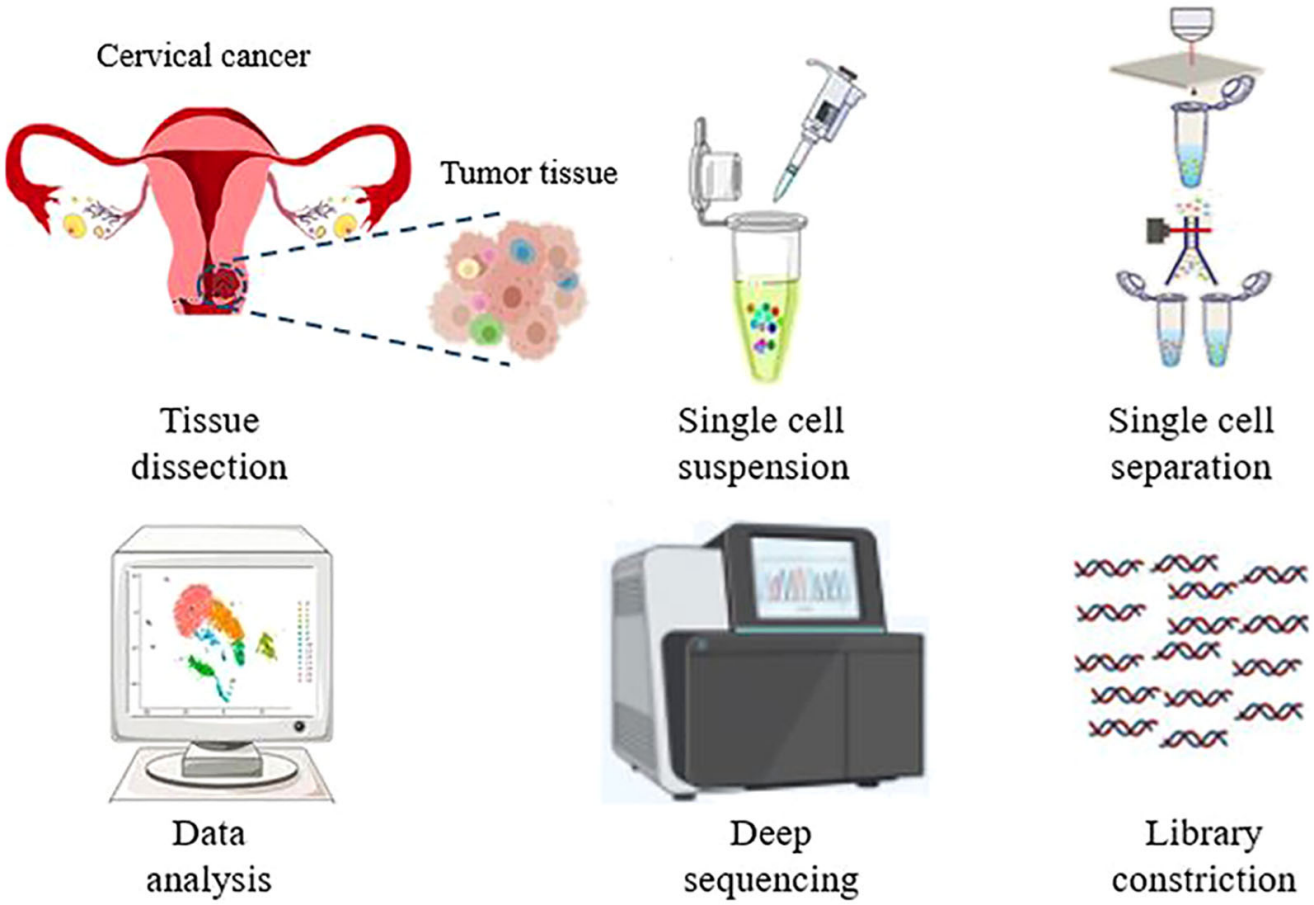
Schematic representation of a typical workflow for single-cell RNA sequencing. Starting from dissociating target cells from the tissue, single cell separation, library construction, single-cell sequencing, and data analysis. Created with BioRender.com.

In addition to proteomics, RNA sequencing (RNA-seq) is essential for advancing our understanding of CC and CCSCs. Schematic diagram of single-cell sequencing is illustrated in **Figure 5**. RNA sequencing enables the identification of molecular heterogeneity, discovery of novel biomarkers, characterization of stemness and differentiation, and uncovering treatment resistance mechanisms.

The insights gained from RNA-seq studies have the potential to improve early detection, risk stratification, and the development of targeted therapies for cervical cancer patients. In recent published paper by Liu and colleagues (134), researchers conducted single-nucleus RNA sequencing (snRNA-seq) analysis on 42,928 nuclei from stage-I cervical cancer (CCI) patients and 29,200 nuclei from stage-II cervical cancer (CCII) patients. They used bioinformatics tools to compare cell heterogeneity and functions, and also performed label-free quantitative mass spectrometry-based proteomic analysis. The proteome profiles of CCI and CCII patients were compared and integrated with the snRNA-seq data. The results revealed that immune response relevant signaling pathways were suppressed in immune cells of CCII patients compared to CCI patients. However, signaling associated with cell and tissue development was enriched in CCII patients, as well as metabolism for energy production indicated by the upregulation of genes associated with mitochondria. The quantitative proteomic analysis supported these findings, showing an abundance of proteins promoting cell growth and intercellular matrix development in the tumor microenvironment of CCII patients (134). The study identified interferon-α and γ responses as the most activated pathways in many cell populations of CCI patients. Additionally, several collagens, including COL4A1, COL5A1, COL12A1, and COL4A2 were significantly upregulated in the CCII group, suggesting their potential role in diagnosing CC progression. Furthermore, a novel transcript called AC244205.1 was found to be highly upregulated in CCII patients, indicating its possible involvement in CC, warranting further investigation (134).

Despite numerous studies being carried out on CCSC-targeting therapies, several limitations still exist that are difficult to overcome. CCSCs are typically present in very low numbers within tumors. In addition, CCSC-targeted therapy may harm normal stem/progenitor cells and hinder the regeneration of normal tissues, resulting in tissue and/or organ dysfunction. Another challenge in studying CCSCs is their rarity and heterogeneity. Nevertheless, recent advances in proteomic techniques have made it probable to ascertain and quantify proteins in complex mixtures, such as cancer cell populations. Proteomics is a powerful tool for studying CCSCs because it enables researchers to identify and compare protein expression profiles of these cells with those of non-cancerous cervical stem cells and other cell types (104, 118, 135).

## 6 Conclusion and outlook

Proteomics-based approaches have played a crucial role in the discovery of biomarkers for the diagnosis, prognosis, and treatment of CC. As opposed to currently employed techniques like Pap smears and HPV testing, the introduction of proteomic biomarkers may allow for earlier detection and better management of CC. Mass spectrometry-based techniques, coupled with advanced data analysis algorithms, allow for the high-throughput screening of large numbers of samples, facilitating the discovery of novel biomarkers with high sensitivity and specificity. Proteomic profiling can also reveal alterations in protein expression and modifications that can aid in patient stratification, disease classification, prognosis prediction, and personalized treatment strategies. It also sheds light on the molecular mechanisms underlying drug resistance and facilitates the development of targeted therapies. The commercialization of proteomic technologies offers opportunities for collaborations between academia and industry, leading to the development of innovative diagnostic tools and therapeutic interventions. Integrating proteomics with other omics technologies, along with advances in bioinformatics and data analysis, can help identify robust biomarker panels with increased predictive power, as well as provide a more comprehensive understanding of the molecular mechanisms underlying CC development and progression. In addition, proteomic studies specifically targeting CCSCs can reveal the proteomic changes associated with these cells and identify CCSC-related biomarkers. However, there are still challenges to overcome. Standardization of protocols and data analysis methods is necessary to ensure reproducibility and comparability of results across different laboratories and studies. Additionally, the integration of proteomic methods into routine clinical practice requires further validation and clinical trials to establish their reliability and utility.

Continued advancements, standardization efforts, and clinical validation will further enhance the translation of proteomics into clinical practice. Personalized risk assessment, early detection, and patient-tailored drug selection are just a few of the ways in which proteomics-based testing has the potential to dramatically improve CC management.

## Author contributions

AJ: Conceptualization, Investigation, Project administration, Writing – original draft, Writing – review & editing. MF: Writing – review & editing. MA-A: Writing – original draft. AM-T: Writing – original draft. MY: Writing – review & editing. MR-T: Supervision, Writing – review & editing.
